# Prevalence and correlates of depression among Thai university students: nationwide study

**DOI:** 10.1192/bjo.2025.21

**Published:** 2025-03-19

**Authors:** Nucharapon Liangruenrom, Mohsen Joshanloo, Wannee Hutaphat, Sirinan Kittisuksathit

**Affiliations:** 1 Institute for Population and Social Research, Mahidol University, Salaya, Thailand; 2 Department of Psychology, Keimyung University, Daegu, South Korea

**Keywords:** Depression, students, happy university, mental health, HAPPINOMETER

## Abstract

**Background:**

Depression is a growing mental health concern among university students worldwide, including in Thailand. Studies show that between 17 and 40% of Thai university students experience depressive symptoms, although these studies often focus on specific groups and use different measures.

**Aims:**

This study aimed to investigate the prevalence and associated factors of depressive disorders among university students across Thailand.

**Method:**

Data were collected with the HAPPINOMETER and the Patient Health Questionnaire-9 scale as part of a nationwide cross-sectional survey. A total of 14 621 students from 33 universities participated in the survey. A series of binary logistic regression analyses were performed to examine the effect of sociodemographic characteristics and health behaviours on the presence of depression.

**Results:**

The results revealed that 31.4% of students experienced depressive symptoms, with 14.2% having major depressive disorder. The students who identified as non-binary had the highest odds of having depression (adjusted odds ratio 2.10, 95% CI 1.7–2.6, *P* < 0.001). Other specific subgroups were also found to be particularly vulnerable, including women, fourth-year students and those studying in Bangkok, without part-time jobs, living alone and engaging in risky health behaviours like smoking, heavy drinking and poor diet.

**Conclusions:**

The findings underscore the need for comprehensive mental health support and targeted interventions within Thai universities, especially for at-risk subpopulations. Leveraging the existing collaborative networks among Thai universities presents a unique opportunity to mount a coordinated effort in developing and implementing comprehensive mental health strategies tailored to the needs of this vulnerable population.

The World Health Organization highlights that globally, around 3.8% of the world’s population experiences depression, which can lead to suicide, the world’s fourth leading cause of mortality among individuals aged 15–29 years.^1^ Over 80% of people living with mental disorders reside in low- and middle-income countries (LMICs), including Thailand.^2^ Reflecting global trends, Thailand has seen a significant rise in the prevalence of depression. According to the Department of Mental Health, Ministry of Thai Public Health, approximately 2.9 million Thai people aged 15 years and above were diagnosed with depression in 2023, increasing from 1.3 million in 2015.^3^ Key drivers, such as rapid urbanisation, socioeconomic inequalities and the impact of the COVID-19 pandemic, have notably contributed to this increase in depression, both in Thailand and other LMICs.^2,4^

University students in Thailand face heightened vulnerability to depression, driven by academic pressures, financial stress and the challenges of transitioning to adulthood.^5^ These stressors are heightened by limited access to mental health resources, stigma associated with seeking help and low mental health literacy. Research indicates that many Thai students struggle to identify symptoms of mental health conditions and are unaware of available resources or effective coping strategies.^4,6,7^ For example, Musumari et al found that only around 6% of university students in Thailand had a history of using mental health services, reflecting significant barriers to awareness and accessibility.^6^ These vulnerabilities are evident in studies reporting the prevalence of depression among Thai university students to be between 17 and 40%.^6,8–11^

The COVID-19 pandemic has further exacerbated these challenges, with a significant proportion of Thai students reporting depression (43.4%), anxiety (36.7%) and stress (29.7%) during the pandemic.^12^ Factors contributing to these heightened psychological effects included difficulties adapting to online learning, a lack of practical skills, low confidence in abilities, diminished social interactions and concerns over academic delays.^12^ Additionally, a recent study on university students in Thailand after the pandemic highlighted the interplay between stress, depression and public stigma, emphasising the need for targeted interventions to address these interconnected factors.^13^

## The role of universities in addressing student depression and advancing sustainable development goals

Universities represent a unique microcosm that provides a robust framework for addressing mental health issues such as depression among students. This environment offers several advantages, including a concentrated population of young adults, access to educational and health resources, and the ability to foster community and collaboration. With these unique opportunities, universities can create supportive academic environments through initiatives such as peer support programmes, on-campus mental health services and interventions targeting key stressors such as academic pressures, financial challenges and social adaptation.^5^ These efforts can not only alleviate immediate stress, but also increase mental health literacy and accessibility for students.^4,6,7^

In Thailand, universities have expanded these efforts by forming collaborative networks to address health challenges faced by their students. For example, the Thai University Network for Health Promotion Network and Happy University Network actively prioritise mental health through joint initiatives and knowledge-sharing platforms. A notable example of such a concerted effort is the recent Thai Healthy University Network Conference, titled ‘Building a Culture of Health Promotion: Fostering Mental Health Awareness and Support on Campus’. Such collaborative efforts amplify the impact of individual universities by leveraging shared resources and expertise to integrate comprehensive mental health programmes and advocate for policies that prioritise student well-being.

Moreover, universities worldwide share a joint mission to promote academic excellence and prepare graduates to contribute positively to society. This shared purpose aligns closely with addressing significant global challenges, including the Sustainable Development Goals (SDGs) set by the United Nations. The study of depression among university students is particularly relevant to several SDGs, notably those focusing on health, education and well-being.^4^ By addressing these issues, universities not only enhance individual health outcomes, but also contribute to broader societal progress.

## Addressing the gap: comprehensive analysis of depression among Thai university students

Despite the recognition of depression as a significant issue among Thai university students, there remains a lack of comprehensive studies that explore the prevalence and multifaceted correlates of depression in this population. To fill this gap, this study aims to investigate the prevalence and associated factors of depressive disorders among university students across Thailand. Previous studies have often focused on specific student groups, recruited small sample sizes or were confined to single universities, limiting geographical and demographic coverage. Additionally, these studies employed varying assessment measures, complicating comparisons and generalisation across the student population. The present study seeks to address these limitations by providing a more inclusive analysis of depression among university students across Thailand, using the standardised Patient Health Questionnaire-9 (PHQ-9) to ensure consistency in measurement and comparability of findings.^14,15^ Such an approach offers comprehensive and standardised data and strengthens a collaborative university network that can influence mental health policy changes at organisational, national and international levels.

## Method

### Study design and participants

The study used cross-sectional data from the health and well-being survey of university students using the HAPPINOMETER, a nationwide project to assess and improve university student well-being. This survey was distributed to member universities of the Happy University network throughout Thailand during the 2022 academic year. At the time of the survey, there were 33 member universities from all regions of Thailand. Current students from these universities were invited to participate voluntarily. The invitation process varied by university, with most students receiving an email through their university system containing a link to the online survey. In cases where paper-based questionnaires were preferred, these were distributed to universities upon request. No specific random sampling method was applied; instead, the survey was open to all students within the participating universities, aiming to maximise coverage and representation across diverse regions and demographics. This study included students from all years and degree programmes.

A total of 19 731 students participated in the survey. After excluding participants with missing data (*n* = 5110), the final sample size was 14 621 participants. Although no formal power analysis was conducted before data collection, the large sample size provides a solid foundation for multivariable analyses and meaningful exploration of depression prevalence and associated factors among university students. However, it is important to note that this sample may not fully represent the broader Thai university student population, because of the voluntary nature of participation and the inclusion of member universities only. Despite these limitations, this study includes one of the largest samples of university students in Thailand to date.

### Study instruments

This study is based on the HAPPINOMETER survey, a self-administered questionnaire developed in 2008 to monitor and evaluate quality of life and happiness across multiple dimensions, including physical and mental health, family life, environment and living conditions, morality, financial well-being, engagement and work–life balance among working adults.^16^ The questionnaire was developed and refined through a literature review and consultation meetings with experts in related fields. Over time, the HAPPINOMETER has been used across several sectors, including higher education. Currently, there are two versions of the questionnaire tailored both for working adults and university students. Both versions were pretested; the adult version had 200 workers from 50 workplaces, and the student version had 30 university students. Each demonstrated strong reliability, with Cronbach’s alpha values of 0.969 and 0.826, respectively, ensuring high internal consistency. The student version, employed in this study, was adapted from the adult version to address the unique circumstances and challenges of university life. These adaptations included a shift from work-life balance to adjustment to campus living, peer relationships and opportunities for extracurricular involvement.

For assessing mental health in the student population, the survey included the PHQ-9, a nine-item self-report scale. In Thailand, the PHQ-9 has been incorporated into the Clinical Practice Guideline of Major Depressive Disorder for General Practitioners (CPG-MDD-GP), serving as a standard tool to assess symptoms of depression, anxiety and stress. This study employed the translated and validated Thai version of PHQ-9, which has acceptable psychometric properties for screening depression (e.g. Cronbach’s alpha = 0.79).^14^ This study used only PHQ-9 results from the survey as an outcome variable, and sociodemographic information, weight status and health behaviours as independent variables.

### Outcome variable

The primary outcome of the study was depression, as measured by the nine-item PHQ-9 scale. The scale was used to indicate feelings or symptoms that students had experienced in the past 2 weeks. To measure the severity of depression symptoms, each item was scored from 0 to 3 (0 = not at all, 1 = some days (1–7 days), 2 = often (> 7 days) and 3 = every day). The total sum of nine items (between 0 and 27) was categorised into five groups: 0–4 = no or minimal depression, 5–9 = mild depression, 10–14 = ‘moderate depression, 15–19 = moderate to severe depression and 20–27 = severe depression. These categories are based on validated clinical guidelines for depression severity, widely accepted in research and clinical contexts.^15^

To assess the diagnostic status of depression, students’ responses were categorised into three groups: no depression, other depressive disorder and “major depressive disorder. Students were considered to have major depressive disorder if five or more items were rated at least 2 (often), except for the last item on suicidal ideation, which was counted if was rated at least 1 (some days). Moreover, one of the symptoms had to correspond to little interest in doing things or feeling depressed. For other depressive disorders, students were included in this category if two to four items were rated at least 2 (often), and the last item was rated at least 1. One of the symptoms also had to correspond to little interest in doing things or feeling depressed. Major depressive disorder and other depressive disorders were also combined to indicate the presence of depression. The remainder would be classified as ‘no depression’.

### Independent variables

The survey included sociodemographic information, weight status and health behaviours of the participants. These characteristics were used as independent variables, including gender (male, female and non-binary), year of study (first, second, third, fourth and higher years), region (Bangkok, Central, East, West, North, North-East and South), university campus in hometown (yes, no), part-time employment (yes, no) and living arrangement (living alone, living with family, living with friend and living with partner). Body mass index (BMI) was calculated based on students’ self-reported weight and height. Five BMI categories were defined: underweight (<18.5 kg/m^2^), normal (18.5–22.9 kg/m^2^), overweight (23–24.9 kg/m^2^), obese (25–29.9 kg/m^2^) and morbidly obese (>30 kg/m^2^).

Students also reported the frequency or intensity of their health behaviours, including smoking; alcohol consumption; consumption of foods high in sugar, salt and fat; consumption of fruits and vegetables; and physical activity. They would rate these behaviours on a scale of 1–5, with 1 indicating maximum risk and 5 indicating no risk. The score for each behaviour was recoded into high risk (score of 1 or 2), moderate risk (score of 3) and good behaviour (score of 4 or 5).^16^

### Data analysis

Descriptive statistics were used to describe the basic characteristics of the sample. Percentages and the chi-squared test were used to assess the statistical significance (at *P* < 0.05) of participants experiencing depression. Residual analysis was also conducted to examine the specific contributions of cells to the significant results. Standardised residuals greater than ±1.96 were considered significant at *P* < 0.05. In addition to the significance level, the Phi and Cramer’s V effect sizes were calculated to measure the magnitude of the association. Phi and Cramer’s V are measures of association for categorical variables in contingency tables. Phi is used explicitly for 2 × 2 tables, whereas Cramer’s V is used for larger tables. Both measures range from 0 to 1.

Given that all of the Phi and V values in this study are below 0.10, these effects can be considered weak.^17^ These calculations were performed for the entire sample and specific sociodemographic characteristics and health behaviours. A series of binary logistic regression analyses were then performed to examine the effect of the predictor variables on the presence of depression. Odds ratio, adjusted for gender, study year, region, part-time employment, living arrangement, BMI category, smoking, alcohol consumption, high-sugar dietary intake, high-salt dietary intake, high-fat dietary intake, fruit and vegetable intake, and exercise were calculated, along with their 95% confidence interval. All analyses were performed in R version 4.3.1 for Windows (R Foundation for Statistical Computing, Vienna, Austria; see https://cran.r-project.org/ ), using the ‘dplyr’, ‘haven’, ‘psych’ and ‘rcompanion’ packages.

### Ethical approval

The authors assert that all procedures contributing to this work comply with the ethical standards of the relevant national and institutional committees on human experimentation and with the Helsinki Declaration of 1975, as revised in 2013. All procedures involving human participants were approved by the Institutional Review Board of the Institute for Population and Social Research, Mahidol University (COA number IPSR-IRB-2022-171). All participants were informed about their rights before they started the survey. They knew the survey was anonymous and signed the consent form before taking part in the survey.

## Results

### Demographic characteristics

A total of 14 621 Thai university students from 33 universities across Thailand were included in the study. The mean age of the participants was 20.75 years (s.d. ± 2.97). The majority of the sample was female (71.28%) and lived with their family (64.92%). The first-year students (32.12%) were more represented in the survey than their seniors. Approximately half of the students were studying at a university outside their hometown (55.16%) and were not working part time (50.20%). Fewer than half of the students in the sample had a normal weight status (44.77%), and the remainder were underweight (22.61%), overweight (10.89%), obese (13.22%) or morbidly obese (8.50%) (see Table 1).

**Table 1:** Demographic characteristics and prevalence of depression

Variable	Total sample n (%)	No depression n (%)	Experienced depression n (%)	Standardised residual^a^	P-value
Total	14 621 (100)	10 032 (68.61)	4589 (31.39)		
Gender					
Male	3797 (25.97)	2814 (74.11)	983 (25.89)	8.48	<0.001***
Female	10422 (71.28)	6992 (67.09)	3430 (32.91)	6.26	
Non-binary	402 (2.75)	226 (56.22)	176 (43.78)	5.43	
Study year					
First year	4695 (32.12)	3308 (70.46)	1387 (29.54)	3.30	0.008**
Second year	4007 (27.41)	2732 (68.18)	1275 (31.82)	0.69	
Third year	3345 (22.88)	2275 (68.01)	1070 (31.99)	0.85	
Fourth year	2358 (16.13)	1580 (67.01)	778 (32.99)	1.83	
Above	213 (1.46)	135 (63.38)	78 (36.62)	1.65	
Region					
Bangkok	2468 (16.88)	1602 (64.91)	866 (35.09)	4.35	<0.001***
Central	3909 (26.74)	2467 (63.11)	1442 (36.89)	8.66	
East	1054 (7.21)	777 (73.72)	277 (26.28)	3.71	
West	997 (6.82)	722 (72.42)	275 (27.58)	2.68	
North	2469 (16.89)	1838 (74.44)	631 (25.56)	6.85	
North-East	3655 (25.00)	2586 (70.75)	1069 (29.25)	3.21	
South	69 (0.47)	40 (57.97)	29 (42.03)	1.91	
University campus in hometown					
Yes	6543 (44.84)	4456 (68.1)	2087 (31.9)	1.23	0.226
No	8049 (55.16)	5558 (69.05)	2491 (30.95)	1.23	
Part-time employment					
Yes	7281 (49.8)	5288 (72.63)	1993 (27.37)	10.42	<0.001***
No	7340 (50.20)	4744 (64.63)	2596 (35.37)	10.42	
Living arrangement					
Living alone	1814 (12.44)	1182 (65.16)	632 (34.84)	3.38	<0.001***
Living with family	9467 (64.92)	6575 (69.45)	2892 (30.55)	2.97	
Living with friend	2285 (15.67)	1595 (69.80)	690 (30.20)	1.33	
Living with partner	1016 (6.97)	653 (64.27)	363 (35.73)	3.09	
BMI category					
Underweight	3303 (22.61)	2223 (67.30)	1080 (32.70)	1.85	0.068
Normal	6541 (44.77)	4548 (69.53)	1993 (30.47)	2.16	
Overweight	1591 (10.89)	1091 (68.57)	500 (31.43)	0.03	
Obese	1932 (13.22)	1338 (69.25)	594 (30.75)	0.66	
Morbidly obese	1242 (8.50)	823 (66.26)	419 (33.74)	1.84	

BMI, body mass index.

a. Standardised residual greater than ±1.96 is considered significant at the *P* < 0.05 level.

**P < 0.01, ***P < 0.001.

### Prevalence of depression by demographic characteristics

Regarding the prevalence of depression, 31.39% of the students reported that they had experienced depressive symptoms in the past 2 weeks, and 14.19% of them had major depressive disorder. Regarding the severity of depression, the majority of students reported mild depression (40.90%), whereas moderate, moderately severe and severe depression were 22.02, 7.68 and 2.98%, respectively (see Table 2). When compared by demographic groups, the prevalence of current depressive symptoms was higher among non-binary students (*P* < 0.001, Cramer’s V = 0.087), those who studied in Bangkok or central Thailand (*P* < 0.001, Cramer’s V = 0.098), those who were not currently employed (*P* < 0.001, Phi = −0.09) and those who lived alone or with a partner (*P* < 0.001, Cramer’s V = 0.042). A lower prevalence of depression was found among first-year students (*P* = 0.008, Cramer’s V = 0.031) (see Table 1).

**Table 2:** Prevalence and severity of depression

Variable	n	%
Depression diagnostic status		
No depression	10 032	68.61
Other depressive disorder	2515	17.20
Major depressive disorder	2074	14.19
Depression severity		
No or minimal	3862	26.41
Mild	5980	40.90
Moderate	3220	22.02
Moderately severe	1123	7.68
Severe	436	2.98

### Prevalence of depression by health behaviours

As shown in Table 3, for the health behaviour variables, students with a high intake of unhealthy foods (high in salt, fat and sugar) and tobacco use reported the highest prevalence of current depressive disorders (43.93, 43.29, 41.83 and 41.93%, respectively) and the lowest prevalence of no depression (56.07, 56.71, 58.17 and 58.07%, respectively). These differences were statistically significant, but with a small effect (*P* < 0.001, Cramer’s V = 0.15, 0.13, 0.13 and 0.074, respectively). Moreover, such healthy behaviour as exercise was significant for the presence of depressive symptoms (*P* < 0.001, Cramer’s V = 0.12). Students who exercised sufficiently (identified as ‘good behaviour’) reported the lowest prevalence of current depressive disorders (23.53%) and the highest prevalence of no depression (76.47%). Similar patterns were found for all other behaviours, in that those who engaged in good health behaviours had the highest absence of depression and the lowest presence of depression. In addition, the presence of depression was significantly different between health behaviour levels; the better the health behaviours that students engaged in, the lower the prevalence of depression (*P* < 0.001).

**Table 3:** Absence and presence of depressive symptoms according to levels of health behaviours

Variable	All sample n (%)	No depression n (%)	Experienced depression n (%)	Standardised residual^a^	P-value
Smoking					
High risk	725 (4.96)	421 (58.07)	304 (41.93)	6.27	<0.001***
Moderate risk	901 (6.16)	537 (59.6)	364 (40.4)	6.02	
Good behaviour	12995 (88.88)	9074 (69.83)	3921 (30.17)	8.94	
Alcohol consumption					
High risk	3468 (23.72)	2235 (64.45)	1233 (35.55)	6.05	<0.001***
Moderate risk	4891 (33.45)	3338 (68.25)	1553 (31.75)	0.67	
Good behaviour	6262 (42.83)	4459 (71.21)	1803 (28.79)	5.85	
High-sugar food intake					
High risk	3361 (22.99)	1955 (58.17)	1406 (41.83)	14.87	<0.001***
Moderate risk	5090 (34.81)	3549 (69.72)	1541 (30.28)	2.12	
Good behaviour	6170 (42.20)	4528 (73.39)	1642 (26.61)	10.63	
High-salt food intake					
High risk	2996 (20.49)	1680 (56.07)	1316 (43.93)	16.59	<0.001***
Moderate risk	5160 (35.29)	3549 (68.78)	1611 (31.22)	0.32	
Good behaviour	6465 (44.22)	4803 (74.29)	1662 (25.71)	13.17	
High-fat food intake					
High risk	2333 (15.96)	1323 (56.71)	1010 (43.29)	13.52	<0.001***
Moderate risk	5519 (37.75)	3716 (67.33)	1803 (32.67)	2.60	
Good behaviour	6769 (46.30)	4993 (73.76)	1776 (26.24)	12.46	
Vegetable and fruit intake					
High risk	3257 (22.28)	2008 (61.65)	1249 (38.35)	9.71	<0.001***
Moderate risk	5701 (38.99)	3969 (69.62)	1732 (30.38)	2.09	
Good behaviour	5663 (38.73)	4055 (71.61)	1608 (28.39)	6.20	
Exercise					
High risk	10286 (70.35)	6735 (65.48)	3551 (34.52)	12.59	<0.001***
Moderate risk	2121 (14.51)	1604 (75.62)	517 (24.38)	7.52	
Good behaviour	2214 (15.14)	1693 (76.47)	521 (23.53)	8.64	

a. Standardised residual greater than ±1.96 is considered significant at the *P* < 0.05 level.

***P < 0.001.

### Associations of demographic characteristics and health behaviours with the presence of depression

The non-binary students had the highest odds of having depression (2.1 times higher than male students), and female students were 40% more likely to have a depressive disorder than male students (*P* < 0.001). Significantly higher odds of depression were also found for those who were in their fourth year of study (compared with those in their first year), did not have a part-time job (compared with those who had a part-time job), and lived alone or with a partner (compared with those who lived with family). Participants who studied in the East, North, North-East and West of Thailand had lower odds of having depressive symptoms than those who studied in Bangkok, by 36, 33, 29 and 23%, respectively. Studying on a campus located in another area than students’ hometown also significantly reduced the odds of depression by 9%. In terms of health behaviours, students who engaged in healthy behaviours had lower odds of developing a depressive disorder than those who engaged in risky health behaviours (see Table 4).

**Table 4:** Presence of depression: associations with demographic characteristics and health behaviours of university students

Variable	Adjusted odds ratio	95% CI	P-value
Gender (reference: male)			
Female	1.40	1.27–1.537	<0.001***
Non-binary	2.10	1.686–2.622	<0.001***
Study year (reference: first year)			
Second year	1.09	0.991–1.201	0.075
Third year	1.06	0.954–1.169	0.291
Fourth year	1.15	1.025–1.284	0.017*
Above	1.19	0.875–1.603	0.264
Region (reference: Bangkok)			
Central	1.10	0.988–1.234	0.081
East	0.64	0.538–0.751	<0.001***
West	0.77	0.649–0.909	0.002**
North	0.67	0.588–0.764	<0.001***
North-East	0.71	0.635–0.802	<0.001***
South	1.49	0.889–2.456	0.125
University campus in hometown (reference: yes)			
No	0.91	0.843–0.983	0.016*
Part-time employment (reference: yes)			
No	1.40	1.3–1.512	<0.001***
Living arrangement (reference: living with family)			
Living alone	1.33	1.189–1.495	<0.001***
Living with friend	0.97	0.875–1.083	0.623
Living with partner	1.19	1.028–1.372	0.019*
BMI category (reference: normal)			
Underweight	1.01	0.923–1.113	0.773
Overweight	1.06	0.934–1.196	0.377
Obese	1.06	0.944–1.189	0.325
Morbidly obese	1.09	0.954–1.251	0.199
Smoking (reference: high risk)			
Moderate risk	0.91	0.741–1.125	0.394
Good behaviour	0.58	0.485–0.683	<0.001***
Alcohol consumption (reference: high risk)			
Moderate risk	0.87	0.783–0.957	0.005**
Good behaviour	0.74	0.671–0.822	<0.001***
High-sugar food intake (reference: high risk)			
Moderate risk	0.76	0.685–0.833	<0.001***
Good behaviour	0.75	0.674–0.825	<0.001***
High-salt food intake (reference: high risk)			
Moderate risk	0.69	0.628–0.766	<0.001***
Good behaviour	0.60	0.537–0.66	<0.001***
High-fat food intake (reference: high risk)			
Moderate risk	0.83	0.747–0.926	0.001**
Good behaviour	0.71	0.637–0.798	<0.001***
Vegetable and fruit intake (reference: high risk)			
Moderate risk	0.79	0.722–0.872	<0.001***
Good behaviour	0.73	0.666–0.808	<0.001***
Exercise (reference: high risk)			
Moderate risk	0.74	0.66–0.826	<0.001***
Good behaviour	0.76	0.679–0.854	<0.001***

Odds ratios are adjusted for all other variables in the table. BMI, body mass index.

*P < 0.05, **P < 0.01, ***P < 0.001.

## Discussion

Depression has become one of the significant mental health challenges worldwide, including in Thailand, especially among university students. This study assessed perceived depression and its associated factors among 14 621 university students from 33 higher educational institutions across Thailand. Our findings reveal that nearly a third of the university students experienced depression in their past weeks, and about 14% of them had major symptoms. Additionally, the severity of depressive symptoms was found to be moderately high or high in more than 10% of the whole sample. Moreover, the present study identifies several specific sub-populations that are particularly vulnerable to depression. These include non-binary students, women, fourth-year students, those studying in Bangkok, those living in their hometowns, students without part-time jobs, those living alone or with a partner, and those engaging in high-risk health behaviours. These results indicate a relatively high prevalence and severity of depression problems among this population and highlight specific subpopulation groups that may be considered a priority for the implementation of mental health services in a university setting.

The prevalence rate of 31.4% of depression among Thai university students shows consistency with the worldwide estimate of 33.6% reported in 2021.^18^ This consistency likely reflects universal stressors faced by university students, such as academic pressures, social challenges and financial situations.^5,18^ Our findings are also consistent with the prevalence reported for South-East Asian countries (31.4%)^4^ and upper-middle-income countries (30.8%),^18^ suggesting that comparable socioeconomic conditions and educational systems may influence mental health outcomes in these regions. Regional variations, however, provide further insights into contextual factors influencing mental health. For example, although the prevalence of depression among Thai students (31.4%) is comparable to that in Malaysia (33.8%),^19^ Thailand’s sociocultural dynamics, such as pervasive mental health stigma, may create unique barriers to early intervention.^7^ Despite similar stressors, the stigma surrounding mental health in Thailand may limit awareness and discourage students from seeking timely support.

It is important to note that our sample may not represent the broader Thai university student population, as data were collected from member universities, and students voluntarily completed the survey. This introduces the possibility of self-selection bias, as students who are more aware of or affected by mental health issues may have been more inclined to participate. Despite being non-representative, this study marks a significant advance over previous Thai-specific studies by including a large, nationwide sample. We surveyed 14 621 students from all regions of Thailand, whereas earlier studies typically involved smaller samples ranging from 475 to 2500 participants.^6,8–11^ Despite the limitations of voluntary participation, our findings offer a broader estimate of depression prevalence among Thai university students. Admittedly, there is a need for studies that use probability sampling techniques to collect nationally representative samples.

Our results also indicate significant differences in the probability of developing depression between gender, study year, employment, region, living arrangement and health behaviours. Consistent with several studies, female students often reported higher levels of depression compared with their male counterparts across various cultural and geographical contexts.^18,20,21^ In Thai society, this disparity may be exacerbated by traditional gender roles and expectations that place additional pressures on female students. Cultural norms often emphasise male dominance and female subservience, reinforcing gender biases and stereotypes that depict women in domestic or passive roles.^22^ These societal expectations intensify the burden on female students to excel academically while conforming to traditional norms. These sociocultural dynamics, combined with factors such as body image concerns and susceptibility to relational stress, likely contribute to the higher prevalence of depression among female students.^23,24^

Our findings also support an interesting gendered pattern of depression, highlighting that non-binary students experience higher levels of depression compared with their cisgender peers.^23,25^ Non-binary populations are particularly vulnerable to depression and compounded stress because of their intersectional identities (e.g. race, gender, sexuality).^25^ They often face internalised homophobia and minority stress, making them more susceptible to depressive symptoms. This underscores persistent issues of discrimination within the Thai education system, affecting non-binary students. A recent scoping review found that 37 of the 115 studies explicitly explored various forms of discrimination in Thai educational settings, such as limited access to services, physical and sexual abuse, bullying and social victimisation based on gender nonconformity.^26^ Other challenges include stigmatising portrayals of transgender populations in school curricula and rigid regulations such as dress codes that enforce binary gender identities.^26^ These systemic pressures significantly contribute to the heightened levels of depression observed among non-binary students.^27^

Depression rates tend to increase as students go through their years of study.^18,21,27^ This study supports this clear trend that students in their earlier study showed less depression. Our finding underscores the impact of academic-related pressures, which accumulate over the years of study and can exacerbate mental health issues over time. In terms of employment, its impact on depression among university students can be mixed. Balancing work and academic responsibilities can present significant time management challenges, potentially contributing to stress and worsening mental health problems. However, our findings suggest that Thai students without part-time jobs were more likely to experience depressive symptoms. This relationship may reflect the central role of employment in students’ lives. For some, part-time work or internships may be perceived as an essential component of the overall student experience, offering opportunities to develop professional skills, gain practical experience and enhance personal growth.^28^ Additionally, students without part-time jobs may include those who have lost jobs or are unable to find work, potentially experiencing financial stress, decreased self-esteem or a sense of exclusion from work-related social interactions. These findings highlight the complex interplay between employment, financial pressures and mental health, warranting further research to explore these dynamics in greater detail.

The geographical location of a university or a student’s hometown can significantly affect mental health. We found that students who attended a university in Bangkok experienced higher rates of depression. Recent research confirms this trend, showing that the prevalence of depression among Thai university students is higher in Bangkok compared with cities in the Northern, North-Eastern and Southern parts of Thailand.^13^ In the context of Thailand, Bangkok’s status as the capital city and educational hub may contribute to unique stressors. The heightened competitiveness and academic pressures in Bangkok likely stem from the concentration of top-tier universities and the higher expectations associated with studying in the city.^29^ Additionally, urban stressors such as traffic congestion, time pressure, limited access to green spaces and environmental pollution can affect overall quality of life and exacerbate mental health challenges for residents of metropolitan cities.^30^

Mental health stigma in Bangkok may also play a critical role. Despite its urban setting, traditional cultural norms in Bangkok might discourage students from seeking help for mental health issues.^7^ Compared with other regions, where community-based support may be more accessible, the independence of urban living could further hinder help-seeking behaviors.^31^ Moreover, the prevalence of depression found in this study was higher among students who lived alone compared with those who lived with family. Previous evidence supports this finding, as lack of immediate emotional support and social isolation can make students’ academic and personal lives more challenging.^20,32^

The present study also adds evidence that smoking and heavy drinking were linked to higher rates of depression. However, as shown in previous studies, the relationship between the two behaviours and depression appears to be bidirectional.^18,21,33^ For example, individuals with depression are more likely to smoke, and smoking can lead to later depressive symptoms. Our findings cannot confirm the direction of the associations as they are based on cross-sectional data. Additionally, we found consistent results of dietary habits and physical activity with previous research, as improvements in diet quality and regular exercise were associated with lower rates of depressive symptoms among university students.^34,35^ These findings highlight the importance of promoting healthy lifestyle behaviours as part of comprehensive mental health strategies in university settings. University health programmes could benefit from incorporating initiatives such as smoking and drinking cessation campaigns, regular light-to-moderate exercise sessions and providing healthier food choices on campus. Previous interventions, especially those implemented in educational settings, have demonstrated effectiveness, particularly among adolescents.^34–36^ Tailoring these programmes to the university environment may enhance their impact on mental health outcomes.

### Strengths and limitations

This study is the first, to our knowledge, to examine the prevalence and correlation of depression among students in higher education settings across Thailand. The relatively large sample size ensured adequate statistical power. The study employed the same PHQ-9 scale in each participating university, allowing for consistent and reliable assessment of depression across the universities. Furthermore, as the PHQ-9 has been widely used in several countries, this may encourage comparative studies in mental health research. However, this study has also some limitations. As we collected data by using a self-administered questionnaire, responses were based on self-assessment of the respondents, and bias from recall period and social desirability may occur. Because of its cross-sectional design, the study’s ability to establish causal relationships between variables is limited; therefore, the identified associations should be interpreted cautiously.

### Recommendations for future directions

Understanding the extent of depression and identifying associated factors is essential for developing targeted interventions and support systems. Addressing mental health issues in educational settings requires a broader range of associations, including psychological, social, cultural and environmental characteristics. These findings suggest the necessity for tailored mental health interventions and support systems that provide for the specific needs of students, particularly those who experience higher rates of depression. Future studies should also align with the SDGs by focusing on enhancing healthy lifestyles and reducing depression among vulnerable groups such as non-binary students. This approach promotes inclusive education and social equity, ensuring all students have the support they need. Future research should explore the impact of targeted interventions and evaluate their effectiveness in reducing depression among university students. Additionally, longitudinal studies could provide valuable insights into the trajectories of mental health challenges throughout the academic journey, enabling proactive and timely support measures.

In conclusion, this nationwide study provides robust evidence of the significant burden of depression among university students across Thailand. Although the observed prevalence of depressive symptoms (31.4%) and major depressive disorder (14.2%) among Thai university students aligns with global trends, these figures are alarmingly high, highlighting the urgency to prioritise mental health support within higher education institutions in Thailand and worldwide. The findings underscore the multifaceted nature of factors associated with depression, encompassing sociodemographic characteristics, living circumstances and lifestyle behaviours. This high prevalence also underscores the urgent need for comprehensive mental health support and effective interventions within Thai universities, particularly among non-binary students, women, fourth-year students, those studying in Bangkok, students without part-time jobs, those living alone and those engaging in high-risk health behaviours.

The relatively high prevalence of depressive symptoms among Thai university students revealed in this study necessitates an urgent, multifaceted and collaborative response from higher education institutions nationwide. Leveraging the existing collaborative networks among Thai universities presents a unique opportunity to mount a coordinated effort in developing and implementing comprehensive mental health strategies tailored to the needs of this vulnerable population. Such a strategy should encompass a multipronged approach, including initiatives aimed at destigmatising mental health issues through psychoeducational programs, ensuring access to professional counselling and therapy services, fostering peer support networks and actively promoting healthy lifestyles through campaigns that encourage regular physical activity and nutritious dietary habits. By synergising their resources and leveraging their collective expertise, Thai universities can create a robust framework that addresses the multidimensional aspects of mental health, ultimately safeguarding the well-being and enabling the personal and academic flourishing of their student communities.

## Data Availability

The data that support the findings of this study are available from the corresponding author, N.L., upon reasonable request.
